# Childhood maltreatment predicts subsequent anxiety symptoms among Chinese adolescents: the role of the tendency of coping styles

**DOI:** 10.1038/s41398-021-01463-y

**Published:** 2021-06-02

**Authors:** Lan Guo, Wanxin Wang, Wenyan Li, Meijun Zhao, Ruipeng Wu, Ciyong Lu

**Affiliations:** 1 Department of Medical Statistics and Epidemiology, School of Public Health, Sun Yat-sen University, 510080 Guangzhou, People’s Republic of China; 2 Guangdong Provincial Key Laboratory of Food, Nutrition and Health, Sun Yat-sen University, 510080 Guangzhou, People’s Republic of China

**Keywords:** Psychiatric disorders, Scientific community

## Abstract

Childhood maltreatment may have an influence on anxiety symptoms and coping styles. This longitudinal study aimed to estimate the prospective associations between different types of childhood maltreatment and anxiety symptoms among Chinese adolescents, with a particular focus on investigating whether these associations vary by the tendency of coping styles. Data were from the Longitudinal Study of Adolescents’ Mental and Behavioral Well-being Research. The baseline sample included 1957 participants (response rate: 99.03%) and followed up at 1-year later (*n* = 1836, retention rate: 93.8%). Anxiety symptoms, childhood maltreatment, the tendency of coping styles, morning cortisol level, depressive symptoms, self-esteem, and other demographics were measured. Overall, the mean age of the baseline students was 13.6 (SD: 1.5) years. The final results showed that childhood emotional abuse (unstandardized β-estimate = 0.13, 95% CI = 0.07–0.18), physical abuse (unstandardized β-estimate = 0.08, 95% CI = 0.01–0.16), and sexual abuse (unstandardized β-estimate = 0.17, 95% CI = 0.04–0.29) were positively associated with anxiety symptoms at follow-up after adjusting for significant covariates at baseline. Additionally, the stratified analyses demonstrated that only among students with negative coping styles, childhood emotional abuse, physical abuse, and sexual abuse were associated with subsequent anxiety symptoms; the differences between the positive and negative coping style strata were significant (*P* < 0.05). Childhood maltreatment appears to be a predictor of anxiety symptoms among adolescents, and the tendency of coping styles may have a moderating role in these longitudinal associations. The efforts to prevent anxiety symptoms are recommended to be focused on adolescents with the experience of childhood maltreatment and negative coping styles.

## Introduction

Childhood maltreatment, defined as any form of physical, emotional, or sexual abuse and neglect that occurs to those under 18 years of age, is thought to affect one in three children globally^1^. It is well described that childhood maltreatment can have long-term effects on health through the lifespan and is associated with the development of mental and physical well-being in adolescence^2^. A recent systematic review found that 20.0% of Chinese adolescents had suffered childhood physical abuse, 30% had suffered emotional abuse, 12% had suffered sexual abuse, 47% had suffered physical neglect, and 44% had suffered emotional neglect, respectively^3^; indicating that childhood maltreatment has also been a major public health problem among Chinese adolescents.

Adolescence is a transitional and developmental period characterized by tremendous physical maturation, social role changes, and psychological development^4^. Moreover, there are considerable changes in the stress-regulatory regions (e.g., prefrontal cortex and hippocampus) during adolescence^5^. Exposure to childhood maltreatment may make adolescents especially susceptible to internalizing psychological difficulties (e.g., symptoms of anxiety or depression). Anxiety symptoms are among the most prevalent internalizing psychological problems among adolescents, resulting in considerable distress to the individual (e.g., impaired school functioning or developing into anxiety disorders) and a substantial burden to society^6^. Previous evidence has suggested that maltreated children were at an increased risk of developing anxiety symptoms and full-blown anxiety disorders in adolescence and adulthood^7,8^. Although some maltreated children had experienced multiple types of childhood maltreatment, current recommendations indicated that different types of childhood maltreatment represented various maltreatment domains and have distinct influences on developmental consequences^9^. For example, deprivation (e.g., physical or emotional neglect) can adversely affect the executive function and learning performance^10^, while threatening contexts (e.g., physical, emotional, or sexual abuse) may disturb emotion regulation and threat responsiveness^11^. However, there is a lack of longitudinal studies addressing the potential associations between each specific type of childhood maltreatment and anxiety symptoms in adolescents. Additionally, morning cortisol level, depressive symptoms, and self-esteem have been reported to be associated with anxiety symptoms among children and adolescents^12–14^. However, there is also a paucity of studies considering the influences of morning cortisol level, depressive symptoms, and self-esteem on the associations between childhood maltreatment and anxiety symptoms.

Moreover, the correlation between coping styles and anxiety symptoms has also received considerable attention. Coping refers to the cognitive and behavioral strategies to reduce, manage, or tolerate the specific internal and/or external demands of stressful situations^15^. Although the broad categorization of coping styles is probably better known, coping styles are most commonly classified into positive coping (i.e., problem-focused coping or engagement coping) and negative coping styles (i.e., emotion-focused coping or disengagement coping)^16^. Previous studies reported that positive coping styles were negatively associated with anxiety symptoms, while negative coping styles might elevate the risk of developing anxiety symptoms^17^. Additionally, prior studies also reported that maltreated children were more likely to adopt avoidant negative coping styles to respond to stressors than their counterparts who did not suffer childhood maltreatment^18^. However, it is not clear the role of coping style in the association between childhood maltreatment and subsequent anxiety symptoms, and the interactions between childhood maltreatment and coping styles contribute to subsequent anxiety symptoms.

Therefore, we conducted this longitudinal study aims to comprehensively test (1) the association between each type of childhood maltreatment and subsequent anxiety symptoms, (2) the interaction effects of childhood maltreatment and coping styles on anxiety symptoms, and (3) the effects of childhood maltreatment on subsequent anxiety symptoms for adolescents with different coping styles.

## Materials and methods

### Study design and participants

Data were from the Longitudinal Study of Adolescents’ Mental and Behavioral Well-being Research in Guangzhou, China (Registration No. ChiCTR1900022032). A multi-stage, stratified cluster, random sampling method was utilized in this study. The sampling and data collection procedures were as follows: in stage 1, six middle schools and three high schools in four districts of Guangzhou, considering the representativeness of the schools across the districts, convenience for the data collection, and previous study collaboration. In stage 2, to avoid loss of follow-up due to students going on for further study, only 7th graders of the middle schools (also can be called students of junior grade one) and 10th graders of the high schools (equal to students of senior grade one) were included in this study. Two classes were randomly selected from the 7th grade and 10th grade within the selected schools, respectively. All students who were available in the selected classes were invited to participate voluntarily. A total of 1957 participants (11–18 years of age) were interviewed at baseline (April to July 2019; response rate: 99.03%) and followed up at 1-year later (*n* = 1836, retention rate: 93.8%). The self-reported questionnaires were blind to teachers to protect the privacy of the students. After the study had been fully explained in detail, written informed consent was obtained from each participant and one of the student’s legal guardians. The study obtained ethical approval from the Sun Yat-sen University, School of Public Health Institutional Review Board (Ethics Number: L2017060).

### Measures

#### Anxiety symptoms

Anxiety symptoms were assessed by the Generalized Anxiety Disorder Scale-7 (GAD-7) in Chinese^19^. The GAD-7 scale has been validated and extensively utilized in Chinese studies with satisfactory psychometric properties^20^, and the Cronbach’s alpha was 0.89 with the current sample. The respondents were asked to rate the frequency of seven anxiety symptoms during the last two weeks, with the response options given on a 4-point scale as follows: 0 = not at all, 2 = several days, 3 = more than half the days, and 4 = nearly every day. The seven items’ total score ranges from 0 to 21, with higher scores indicating more severe anxious symptomatology.

#### Childhood maltreatment at baseline

A history of childhood maltreatment was measured by the Childhood Trauma Questionnaire-Short Form (the CTQ-SF) in Chinese^21,22^, which has been shown to have good reliability and validity in Chinese adolescent groups^23^, and had a high internal consistency (Cronbach’s alpha = 0.86) in the current sample. The CTQ-SF consists of five subscales that assess childhood maltreatment types, including physical neglect, emotional neglect, emotional abuse, physical abuse, and sexual abuse. Each subscale contains five items about experiences that occur in childhood, and the responses are given on a 5-point scale as follows: 1 = never, 2 = rarely, 3 = sometimes, 4 = often, and 5 = very often. Each of the five CTQ-SF subscale scores ranged from 5 to 25, and higher scores suggested more serious maltreatment experiences. In the present study, we described the subscale scores and used the overall CTQ-SF score (a sum score over all items) in the analyses.

#### The tendency of coping style at baseline

Coping style was measured by the Simplified Coping Style Questionnaire (SCSQ)^24^, which was developed by Xie et al. based on the Ways of Coping Questionnaire by Folkman and Lazarus according to Chinese culture and language^25^. The SCSQ has been validated and widely used in Chinese adolescents^26^, and the Cronbach’s alpha was 0.83 with the current sample. The SCSQ includes two dimensions that access the positive coping style and negative coping style. The respondents were asked to rate each item on a 4-point scale (0 = never, 1 = seldom, 2 = sometimes, and 3 = often) based on the frequency they adopted the positive or negative coping style in their daily life, with higher scores indicating greater positive/passive coping. In the present study, the tendency of coping style was assessed by the formula: Tendency of coping style = standard score of positive coping styles − standard score of negative coping style; moreover, the standard score was yielded by *Z*-transforming the mean and standard deviation (SD) of the positive/negative coping style^27^. If the differential value was greater than 0, it indicated that the individual generally adopted a positive coping style and vice versa.

### Other covariates

#### Morning cortisol level

The blood sample of the students was drawn from 7:00 to 10:00 a.m. The morning serum total cortisol level was assayed with the competitive chemiluminescent microparticle immunoassay using the Abbott Architect i2000SR system (Abbott Laboratories, Abbott Park, IL).

#### Depressive symptoms and self-esteem

Depressive symptoms were assessed by the Center for Epidemiologic Studies Depression Scale (CES-D) in Chinese, which has been validated and extensively utilized in Chinese populations^28^. Self-esteem was assessed by the Rosenberg Self-Esteem Scale (RSES) in Chinese, which consists of ten items (six positive-direction and four negative-direction questions) associated with overall feelings of self-worth or self-acceptance^29^.

#### Demographic characteristics

Demographic factors included age, sex (1 = boys, 2 = girls), household socioeconomic status (HSS; 1 = excellent or very good, 2 = good, and 3 = fair or poor), living arrangement (1 = living with both parents, 2 = living with a single parent, and 3 = living with others), classmate relations (1 = good, 2 = average, and 3 = poor), relationships with teachers (1 = good, 2 = average, and 3 = poor), ever smoking a cigarette (1 = yes and 2 = no), and ever drinking alcohol (1 = yes and 2 = no).

### Statistical analysis

First, descriptive analyses stratified by the tendency of coping styles were used to describe the sample characteristics. Continuous and categorical data were reported in the form of proportions and means (SD). Student *t*-tests for continuous variables and chi-square tests for categorical variables were conducted to test the differences of baseline sample characteristics between adolescents with a tendency of positive coping styles and negative coping styles. Second, the generalized linear mixed-effects models were performed that accounted for the multi-stage sampling design^30^. Univariable generalized linear mixed-effects models were conducted to explore the potential factors related to anxiety symptoms at baseline and follow-up. The subscales of childhood maltreatment were correlated to each other (Supplementary Table 1; *P* < 0.01) in this study, and current recommendations indicated that different types of childhood maltreatment may have distinct effects on developmental consequences and should be modeled separately^9^. Next, we performed several multivariable generalized linear mixed-effects models. Third, to investigate whether the associations between childhood maltreatment and anxiety symptoms at 1-year follow-up by student’s tendency of coping style, multiplicative interaction items were tested, and *P*-values for the interaction items were calculated. If the interaction items were significantly associated with anxiety symptoms at follow-up, stratified analyses would be conducted. The statistical significance of the differences between the strata was tested by using the 95% confidence interval (CI): $\left( {\beta _1 - \beta _2} \right) \pm 1.96\sqrt {\left( {{\mathrm{SE}}_1} \right)^2 + \left( {{\mathrm{SE}}_2} \right)^2}$. *β*_1_ and *β*_2_ represented the regression coefficients in each stratum, and SE_1_ and SE_2_ were the corresponding standard errors. Fourth, sensitivity analyses were performed by removing all students with a total GAD-7 score > 0 at baseline. Like previous primary analyses, several multivariable generalized linear mixed-effects models were also performed. Additionally, in post hoc sensitivity analyses, we also conducted all models using the total score of CTQ-SF. Regarding the generalized linear mixed-effects models, observations with missing data were eliminated. All statistical analyses were conducted using R software, version 3.6.3 (the R Foundation for Statistical Computing, Vienna, Austria). All statistical tests were two-sided, and a *P*-value of <0.05 was considered statistically significant.

## Results

### Baseline sample characteristics stratified by the tendency of coping style

Table 1 shows baseline sample characteristics stratified by the tendency of coping style. Of the total sample, 50.8% were boys. The mean (SD) age of the total students was 13.6 (SD: 1.5) years, and the mean GAD-7 score of anxiety symptoms at baseline was 3.0 (SD: 6.0). Compared with those with positive coping styles, students who reported negative coping styles were more likely to report having fair HSS, living with a single parent or others, having poor classmate relations, having a poor relationship with teachers, and ever drinking (*P* < 0.05).

**Table 1:** Baseline sample characteristics stratified by the tendency of coping styles at baseline.

Variable (Baseline)	Total	Tendency of coping styles, *n* (%)		*P*-value*
		Positive coping style	Negative coping style	
Total	1957 (100)	928 (47.4)	1029 (52.6)	
Sex				
Boys	994 (50.8)	461 (49.7)	533 (51.8)	0.365
Girls	963 (49.2)	467 (50.3)	496 (48.2)	
Age, mean (SD), year	13.6 (1.5)	13.6 (1.5)	13.5 (1.5)	0.049
HSS				
Excellent	1012 (51.7)	520 (56.0)	492 (47.8)	<0.001
Good	861 (44.0)	379 (40.8)	482 (46.8)	
Fair	77 (3.9)	26 (2.8)	51 (5.0)	
Missing data	7 (0.4)	3 (0.3)	4 (0.4)	
Living arrangement				
Living with both parents	1593 (81.4)	779 (83.9)	814 (79.1)	0.013
Living with a single parent	192 (9.8)	82 (8.8)	110 (10.7)	
Living with others	167 (8.5)	64 (6.9)	103 (10.0)	
Missing data	5 (0.3)	3 (0.3)	2 (0.2)	
Classmate relations				
Good	1662 (84.9)	841 (90.6)	821 (79.8)	<0.001
Average	254 (13.0)	77 (8.3)	177 (17.2)	
Poor	34 (1.7)	8 (0.9)	26 (2.5)	
Missing data	7 (0.4)	2 (0.2)	5 (0.5)	
Relationship with teachers				
Good	1604 (82.0)	838 (90.3)	766 (74.4)	<0.001
Average	307 (15.7)	77 (8.3)	230 (22.4)	
Poor	27 (1.4)	5 (0.5)	22 (2.1)	
Missing data	19 (1.0)	8 (0.9)	11 (1.1)	
Ever smoking a cigarette				
Yes	28 (1.4)	8 (0.9)	20 (1.9)	0.055
No	1918 (98.0)	918 (98.9)	1000 (97.2)	
Missing data	11 (0.6)	2 (0.2)	9 (0.9)	
Ever drinking alcohol				
Yes	639 (32.7)	259 (27.9)	380 (36.9)	<0.001
No	1307 (66.8)	665 (71.7)	642 (62.4)	
Missing data	11 (0.6)	4 (0.4)	7 (0.7)	
CES-D scores, mean (SD)	11.0 (12.0)	8.0 (9.0)	15.0 (13.0)	<0.001
Morning serum total cortisol, mean (SD), nmol/L	223.4 (101.3)	225.0 (100.9)	222.0 (101.8)	0.526
Total RSES scores, mean (SD)	19.4 (5.2)	17.6 (4.6)	21.1 (5.2)	<0.001
Childhood maltreatment, mean (SD)				
CTQ scores of physical neglect	7.2 (2.7)	6.6 (2.2)	7.7 (3.0)	<0.001
CTQ scores of emotional neglect	8.6 (4.9)	7.1 (3.5)	9.9 (5.5)	<0.001
CTQ scores of emotional abuse	7.9 (3.7)	6.9 (2.6)	8.7 (4.3)	<0.001
CTQ scores of physical abuse	6.5 (2.4)	6.0 (1.9)	6.8 (2.8)	<0.001
CTQ scores of sexual abuse	5.4 (1.4)	5.3 (0.9)	5.6 (1.7)	<0.001
Total CTQ scores	35.5 (10.4)	31.9 (7.2)	38.7 (11.8)	<0.001
GAD-7 scores, mean (SD)	3.0 (6.0)	2.0 (4.0)	4.0 (6.0)	<0.001

*HSS* household socioeconomic status, *CES-D* Center for Epidemiology Scale for Depression, *SD* standard deviation, *RSES* Rosenberg Self-Esteem Scale, *GAD* generalized anxiety disorder.

*The chi-square test was used for categorical variables, and the *t*-test was used for age data, CES-D scores, morning serum total cortisol, total RSES scores, childhood maltreatment data, and GAD-7 scores.

### Associations between baseline sample characteristics and anxiety symptoms at baseline and follow-up

Table 2 presents that without adjusting for other variables, ever drinking alcohol, CES-D scores of depressive symptoms, morning serum total cortisol, total RSES scores of self-esteem, CTQ scores of physical neglect/emotional neglect/emotional abuse/physical abuse/sexual abuse, and negative coping style tendency were positively associated with anxiety symptoms at baseline and follow-up. Additionally, baseline GAD-7 scores were also related to anxiety symptoms at follow-up (unstandardized β-estimate = 0.54, 95% CI = 0.50–0.58).

**Table 2:** Factors associated with anxiety symptoms at baseline and follow-up.

Variables (baseline)	Baseline anxiety symptoms (*N* = 1957)	Anxiety symptoms at follow-up (*N* = 1836)
	Unadjusted model, unstandardized β-estimate (95% CI)^a^	Unadjusted model, unstandardized β-estimate (95% CI)^a^
Sex (ref. = girls)		
Boys	−1.09 (−1.25~−0.93)	−1.08 (−1.25~−0.92)
Age (1-age increase)	0.09 (0.04~0.15)	0.14 (0.09~0.19)
HSS (ref. = fair)		
Excellent	−2.29 (−2.69~−1.88)	−1.42 (−1.84~−1.00)
Good	−1.31 (−1.72~−0.91)	−0.64 (−1.06~−0.22)
Living arrangement (ref. = living with others)		
Living with both parents	−0.96 (−1.25~−0.67)	−0.61 (−0.91~−0.31)
Living with a single parent	−0.10 (−0.47~0.29)	−0.04 (−0.42~0.35)
Classmate relations (ref. = poor)		
Good	−3.92 (−4.52~−3.33)	−3.66 (−4.31~−3.01)
Average	−1.66 (−2.29~−1.04)	−2.05 (−2.73~−1.37)
Relationship with teachers (ref. = poor)		
Good	−1.56 (−2.26~−0.86)	−0.31 (−1.08~0.46)
Average	0.68 (−0.05~1.40)	1.24 (0.45~2.03)
Ever smoking a cigarette (ref. = no)	0.68 (0.05~1.32)	−0.10 (−0.80~0.60)
Ever drinking alcohol (ref. = no)	1.21 (1.04~1.37)	1.17 (1.00~1.34)
CES-D scores (1-score increase)	0.32 (0.32~0.33)	0.23 (0.22~0.23)
Morning serum total cortisol (1-level increase)	0.002 (0.001~0.003)	0.002 (0.001~0.002)
Total RSES scores (1-score increase)	0.38 (0.37~0.40)	0.29 (0.28~0.31)
Childhood maltreatment		
Physical neglect (1-score increase)	0.28 (0.21~0.35)	0.16 (0.08~0.23)
Emotional neglect (1-score increase)	0.18 (0.14~0.22)	0.11 (0.07~0.16)
Emotional abuse (1-score increase)	0.50 (0.45~0.55)	0.42 (0.36~0.46)
Physical abuse (1-score increase)	0.44 (0.36~0.52)	0.36 (0.28~0.44)
Sexual abuse (1-score increase)	0.57 (0.44~0.71)	0.48 (0.34~0.62)
Overall childhood maltreatment (1-score increase)	0.15 (0.13~0.16)	0.11 (0.09~0.13)
Tendency of coping styles		
Negative coping style (ref. = positive coping style)	1.95 (1.57~2.34)	1.31 (1.14~1.47)
GAD-7 scores (1-score increase)	NA	0.54 (0.50~0.58)

*HSS* household socioeconomic status, *CES-D* Center for Epidemiology Scale for Depression, *SD* standard deviation, *RSES* Rosenberg Self-Esteem Scale, *GAD* generalized anxiety disorder, *95% CI* 95% confidence interval.

^a^The univariable generalized linear mixed-effects models were performed that accounted for the multi-stage sampling design.

### Associations of childhood maltreatment and the tendency of coping styles with anxiety symptoms at follow-up

As shown in Table 3, after adjusting for significant covariates at baseline, students who reported having suffered emotional abuse (unstandardized β-estimate = 0.13, 95% CI = 0.07~0.18), physical abuse (unstandardized β-estimate = 0.08, 95% CI = 0.01–0.16), and sexual abuse (unstandardized β-estimate = 0.17, 95% CI = 0.04–0.29) had a higher risk of anxiety symptoms at follow-up, respectively (Model 1). Moreover, after further adjusting for the tendency of coping style at baseline, the magnitudes of the estimates for each type of childhood maltreatment with subsequent anxiety symptoms had only minor changes from Model 1 to Model 2.

**Table 3:** Associations of childhood maltreatment and the tendency of coping styles with anxiety symptoms at follow-up.

Variables (baseline)	Anxiety symptoms at follow-up, adjusted model, unstandardized β-estimate (95% CI)^a^	
	Model 1	Model 2
Childhood maltreatment		
Physical neglect (1-score increase)	−0.03 (−0.10~0.04)	−0.03 (−0.10~0.04)
Emotional neglect (1-score increase)	−0.03 (−0.07~0.01)	−0.03 (−0.07~0.01)
Emotional abuse (1-score increase)	0.13 (0.07~0.18)	0.13 (0.07~0.17)
Physical abuse (1-score increase)	0.08 (0.01~0.16)	0.08 (0.01~0.14)
Sexual abuse (1-score increase)	0.17 (0.04~0.29)	0.17 (0.04~0.22)
Overall childhood maltreatment (1-score increase)	0.02 (0.01~0.03)	0.02 (0.004~0.04)
Tendency of coping styles		
Negative coping style (ref. = positive coping style)	0.09 (−0.06~0.24)	NA

Model 1: adjusting for age, gender, HSS, living arrangement, classmate relations, relationships with teachers, smoking, drinking, morning cortisol level, depressive symptoms, self-esteem, and anxiety symptoms at baseline.

Model 2: adjusting for age, gender, HSS, living arrangement, classmate relations, relationships with teachers, smoking, drinking, morning cortisol level, depressive symptoms, self-esteem, anxiety symptoms, and the tendency of coping styles at baseline.

*95% CI* 95% confidence interval, *NA* not available or not applicable.

^a^The multivariable generalized linear mixed-effects models were performed that accounted for the multi-stage sampling design.

### Longitudinal associations between childhood maltreatment and anxiety symptoms at follow-up stratified by the tendency of coping style

First, the multivariable generalized linear mixed-effects models demonstrated that the interaction items (between physical neglect/emotional neglect/emotional abuse/physical abuse/sexual abuse and the tendency of coping style) were significantly associated with anxiety symptoms at follow-up (Table 4; *P* < 0.05). Stratified analyses were conducted separately for students with positive and negative coping styles. Moreover, among students with negative coping styles, those who experienced childhood emotional abuse (unstandardized β-estimate = 0.17, 95% CI = 0.10–0.24), physical abuse (unstandardized β-estimate = 0.13, 95% CI = 0.03–0.23), and sexual abuse (unstandardized β-estimate = 0.19, 95% CI = 0.03–0.34) were at an increased risk of anxiety symptoms at follow-up. Moreover, the differences between the positive and negative coping style strata were statistically significant (*P* < 0.05) (Table 5).

**Table 4:** Associations of interaction items with anxiety symptoms at follow-up.

Interaction items (baseline)	*P*-value for interaction*
	Anxiety symptoms at follow-up
The tendency of coping styles*	
Physical neglect	0.037
Emotional neglect	0.005
Emotional abuse	<0.001
Physical abuse	<0.001
Sexual abuse	0.002
Overall childhood maltreatment	<0.001

*The multivariable generalized linear mixed-effects models were performed that accounted for the multi-stage sampling design.

**Table 5:** Associations between baseline childhood maltreatment and anxiety symptoms at follow-up stratified by the tendency of coping styles.

Variable (baseline)	Anxiety symptoms at follow-up^a^				*P*-value^#^
	Positive coping style		Negative coping style		
	Unstandardized β-estimate (95% CI)	*P*-value	Unstandardized β-estimate (95% CI)	*P*-value	
Physical neglect (1-score increase)	0.04 (−0.06~0.14)	0.478	−0.07 (−0.16~0.02)	0.120	>0.05
Emotional neglect (1-score increase)	0.001 (−0.07~0.07)	0.997	−0.04 (−0.10~2.59)	0.11	<0.05
Emotional abuse (1-score increase)	0.06 (−0.03~0.15)	0.216	0.17 (0.10~0.24)	<0.001	<0.05
Physical abuse (1-score increase)	−0.03 (−0.15~0.09)	0.653	0.13 (0.03~0.23)	0.01	<0.05
Sexual abuse (1-score increase)	0.08 (−0.17~0.32)	0.540	0.19 (0.03~0.34)	0.017	<0.05
Overall childhood maltreatment (1-score increase)	0.005 (−0.03~0.04)	0.780	0.02 (0.01~0.03)	<0.001	<0.05

*95% CI* 95% confidence interval.

^#^The statistical significance of the differences between the strata was tested by using the 95% CI.

^a^The multivariable generalized linear mixed-effects models were performed that accounted for the multi-stage sampling design, and the models were adjusted for age, gender, HSS, living arrangement, classmate relations, relationships with teachers, smoking, drinking, morning cortisol level, depressive symptoms, self-esteem, anxiety symptoms, and the tendency of coping styles at baseline.

### Sensitivity analyses

Sensitivity analyses using all students with a total GAD-7 score > 0 at baseline showed similar associations between childhood maltreatment, the tendency of coping style, and anxiety symptoms at follow-up. After adjusting for significant covariates at baseline, childhood emotional abuse, physical abuse, and sexual abuse were, respectively, associated with an increased risk of anxiety symptoms at follow-up (Supplementary Table 2). Additionally, in post hoc sensitivity analyses, we also reran all models using the overall CTQ-SF score and found similar results.

## Discussion

To our knowledge, this is the first longitudinal study to test the associations between specific types of childhood maltreatment, coping styles, and anxiety symptoms among Chinese adolescents. The main findings of this study showed that after adjusting for significant demographics, morning cortisol level, self-esteem, depressive symptoms, and anxiety symptoms at baseline, adolescents who suffered emotional abuse, physical abuse, and sexual abuse were at an increased risk of anxiety symptoms at 1-year follow-up. The associations of emotional abuse/physical abuse/sexual abuse with subsequent anxiety symptoms had minor changes after further adjusting for the tendency of coping styles. Although the mechanism between childhood maltreatment and anxiety symptoms is still not clear, there have been several possible explanations suggesting that early-life stressor (e.g., childhood abuse) is related to marked long-term changes of brain circuitry regulating stress reactivity, mood, and behavior^31^. First, childhood maltreatment is one of the most typical early-life stressors, which can activate the hypothalamic-pituitary-adrenal axis and persistently alter the autonomic nervous system’s responses to stressors, presumably due to the cortisol, adrenocorticotropic hormone, and corticotropin-releasing hormone hyperactivity^32^. There is growing evidence suggesting that cortisol hypersecretion is a biological risk factor of anxiety symptoms^33^. Second, based on the theories about cognitive/emotional modulation, childhood maltreatment may render detrimental effects across the lifespan by increasing the risk of emotional and cognitive dysregulation, such as emotion suppression, inattention, feelings of nervousness, impaired decision-making, maladaptive coping; all of which could increase the risk of developing anxiety symptoms^34,35^. In the first place, we suggest that childhood maltreatment should be prevented from occurring^36^. Additionally, although the mechanism underlying the associations between childhood maltreatment and subsequent anxiety symptoms remains unclear, we recommended that families, schools, and clinicians should be aware of the adverse effects of childhood maltreatment on adolescents, and to help adolescents with a history of childhood maltreatment minimize or eradicate the deleterious effects of traumatic memories and recalibrate stress response systems through formalized intervention and support^37^.

Moreover, as the previous evidence reported coping styles are associated with anxiety symptoms^17,38^, in our univariable analysis, the tendency of coping style at baseline was significantly associated with subsequent anxiety symptoms. However, after adjusting for other variables, the adjusted significant associations of coping styles with subsequent anxiety symptoms were not shown in the multivariable analysis. Similarly, Horwitz et al.^39^ found that specific problem-focused coping strategies were not independently associated with lower levels of depression or suicidal ideation. Also, preliminary evidence also demonstrated that the experience of childhood maltreatment is associated with the increased possibility of adopting a negative coping style in adolescence^18^. Therefore, the interaction effects of childhood maltreatment and the tendency of coping styles on anxiety symptoms were tested, and a novel finding was that the above-mentioned interaction effects were significantly associated with anxiety symptoms at 1-year follow-up. These results might be related to a possible increase in vulnerability to have subsequent anxiety symptoms following childhood maltreatment among adolescents with the tendency of negative coping styles than those with positive coping styles^17,38^.

Moreover, after stratified by the tendency of coping styles, another novel finding of this study is that only among students with negative coping styles, those who experienced childhood emotional abuse, physical abuse, and sexual abuse were more likely to be involved in anxiety symptoms at follow-up after adjusting for significant demographics, morning cortisol level, self-esteem, depressive symptoms, and anxiety symptoms at baseline. These findings indicated that coping styles could not have a mediator role but maybe a moderator in the associations between baseline childhood maltreatment and subsequent anxiety symptoms among the sampled adolescents. A possible reason for these associations is that coping mechanisms represent adaptive or maladaptive responses to childhood maltreatment and fundamentally buffer or exacerbate the effects of childhood maltreatment on anxiety symptoms^40^. In the present study, negative coping styles may aggravate the adverse health effects of early exposure to emotional/physical/sexual abuse and contribute to stress dysregulation, resulting in the increased risk of anxiety symptoms among adolescents. In contrast, adolescents with positive coping styles may take active problem-solving strategies or seek professional help to counteract the maladaptive outcomes caused by the experience of childhood maltreatment^41^.

Therefore, the implications of this study highlight that prevention or intervention strategies that are helpful to develop positive coping skills might be vital means to combat anxiety symptoms in adolescents with the experience of childhood maltreatment. Moreover, a particular focus should be placed on accessing childhood maltreatment history and coping styles when implementing interventions or treatments on adolescents with current severe anxiety symptoms.

Our study has several limitations. First, the current study draws on a sample from only one city (Guangzhou), so the generalization of the findings may not be applicable to all Chinese adolescents. We would like to expand the study sites in the future study. Second, the study sample only included school students and did not include adolescents absent from schools. However, childhood maltreatment experience or internalizing problems might be more common among those adolescents. Third, although all data were measured by self-report, which may lead to biased reporting of childhood maltreatment experience, coping styles, and anxiety symptoms, self-reports remain a common and accepted method. Fourth, most Chinese middle school students or high school students will transfer to other schools after graduation. To avoid loss of follow-up due to students going on for further study, only students of junior grade one and senior grade one were included in this study. Fifth, although this is a prospective study, the longitudinal sample was limited to 1 year, and we will prolong the follow-up period in the future. Despite these limitations, the primary strength of our study is that, to the best of our knowledge, it is the first longitudinal study among Chinese adolescents to estimate the moderating effects of the tendency of coping styles on the association between specific types of childhood maltreatment and subsequent anxiety symptoms.

In conclusion, this longitudinal study found that baseline childhood maltreatment can predict anxiety symptoms at 1-year follow-up, and the tendency of coping styles may have a moderating role in the longitudinal associations between childhood maltreatment and anxiety symptoms. The study findings may help identify adolescents who may be vulnerable to anxiety symptoms and high-risk adolescents who have suffered from childhood maltreatment, especially those with negative coping strategies. Moreover, although an increased risk of complex symptomatology associated with childhood maltreatment has been described in previous studies, our study findings extended the findings of the mechanisms underlying the associations between childhood maltreatment and anxiety symptoms. Considering the coping skill can be modifiable, positive education and intervention programs that cultivate adolescents’ positive coping styles are recommended to be developed to decrease the risk of having anxiety symptoms, especially among those with the experience of childhood maltreatment.

## Supplementary information

Supplementary Table 1, Supplementary Table 2

## Data Availability

Derived data supporting the findings of this study are available from the corresponding author on request.
