# Supplementary material for: Childhood maltreatment predicts subsequent anxiety symptoms among Chinese adolescents: the role of the tendency of coping styles
Source: Transl Psychiatry. 2021 Jun 2;11:340. doi: 10.1038/s41398-021-01463-y (PMC8172629; doi:10.1038/s41398-021-01463-y)
Supplement: Supplementary file 1 — Supplementary Table 1, Supplementary Table 2 [file 41398_2021_1463_MOESM1_ESM.docx]

**Supplementary table legends**

Supplementary Table 1. Correlations between each type of childhood maltreatment at baseline (N=1,957)

Supplementary Table 2. Sensitivity analyses using all students with a total GAD-7 score > 0 at baseline (N=532)

| **Supplementary Table 1. Correlations between each type of childhood maltreatment at baseline (N=1,957)** | | | | | |
| --- | --- | --- | --- | --- | --- |
| **Correlation (r)** | **Physical neglect** | **Emotional neglect** | **Emotional abuse** | **Physical abuse** | **Sexual abuse** |
| **Physical neglect** | 1 | 0.521* | 0.099* | 0.173* | 0.209* |
| **Emotional neglect** |  | 1 | 0.218* | 0.228* | 0.163* |
| **Emotional abuse** |  |  | 1 | 0.402* | 0.172* |
| **Physical abuse** |  |  |  | 1 | 0.336* |
| **Sexual abuse** |  |  |  |  | 1 |

r means the Pearson’s correlation coefficient.

*: *P*<0.01.

| **Supplementary Table 2. Sensitivity analyses using all students with a total GAD-7 score > 0 at baseline (N=532)** | | | |
| --- | --- | --- | --- |
| **Variable (Baseline)** | **Anxiety symptoms at follow-up*,**  **Adjusted model, unstandardized β estimate (95% CI)** | | |
|  | **Model 1** | **Model 2** | **Model 3** |
| **Childhood maltreatment** |  |  |  |
| Physical neglect (1-score increase) | 0.04 (0.001~0.08) | -0.01 (-0.10~0.07) | -0.02 (-0.10~0.07) |
| Emotional neglect (1-score increase) | 0.06 (0.04~0.08) | 0.05 (-0.02~0.12) | 0.05 (-0.02~0.11) |
| Emotional abuse (1-score increase) | 0.30 (0.26~0.33) | 0.33 (0.16~0.50) | 0.33 (0.16~0.50) |
| Physical abuse (1-score increase) | 0.26 (0.21~0.31) | 0.25 (0.003~0.50) | 0.25 (0.004~0.50) |
| Sexual abuse (1-score increase) | 0.32 (0.05~0.23) | 0.34 (0.05~0.62) | 0.34 (0.05~0.62) |
| Overall childhood maltreatment (1-score increase) | 0.07 (0.05~0.08) | 0.07 (0.02~0.12) | 0.07 (0.02~0.11) |
| **The tendency of coping styles** |  |  |  |
| Negative coping style (Ref.= positive coping style) | 0.27 (0.07~0.48) | 0.07 (0.02~0.12) | NA |

Abbreviations: 95% CI, 95% confidence interval.

*: The generalized linear mixed-effects models were performed that accounted for the multi-stage sampling design.

Model 1: unadjusted models.

Model 2: adjusting for age, gender, HSS, living arrangement, classmate relations, relationships with teachers, smoking, drinking, morning cortisol level, depressive symptoms, and self-esteem at baseline.

Model 3: adjusting for age, gender, HSS, living arrangement, classmate relations, relationships with teachers, smoking, drinking, morning cortisol level, depressive symptoms, self-esteem, and the tendency of coping styles at baseline.
